# Disease resistance traits of IPB-D2 chickens: Characterization of IgY concentrations, Newcastle disease antibody titers, and leukocyte profiles

**DOI:** 10.14202/vetworld.2025.172-177

**Published:** 2025-01-27

**Authors:** Dwi Lestari, Sri Murtini, Niken Ulupi, Asep Gunawan, Isyana Khaerunnisa, Anneke Anggraeni, Cece Sumantri

**Affiliations:** 1 Research Center for Applied Zoology, National Research and Innovation Agency, Bogor, 16911, Indonesia; 2 Division of Medical Microbiology, School of Veterinary Medicine and Biomedical Science, IPB University, Bogor, 16680, Indonesia; 3 Department of Animal Production and Technology, Faculty of Animal Science, IPB University, Bogor, 16680, Indonesia; 4 Research Center for Animal Husbandry, National Research and Innovation Agency, Bogor, 16911, Indonesia

**Keywords:** IPB-D2 chicken, immunoglobulin Y concentration, ND antibody titer, leukocyte profile

## Abstract

**Background and Aim:**

The IPB-D2 chicken, a potential disease-resistant local Indonesian line, has been developed to improve poultry health and productivity for small-scale farmers. This study aimed to characterize the disease resistance traits of IPB-D2 chickens by analyzing key immunological parameters, including immunoglobulin Y (IgY) concentration, Newcastle disease (ND) antibody titers, and leukocyte profiles.

**Materials and Methods:**

A total of 100 IPB-D2 chickens were studied. Blood samples from 21-week-old chickens were collected for analysis of IgY concentrations using indirect enzyme-linked immunosorbent assay, ND antibody titers using the hemagglutination inhibition test, and leukocyte profiling using the Giemsa staining method. Correlations between parameters were determined using Pearson’s correlation analysis in RStudio.

**Results:**

The findings revealed that IPB-D2 chickens exhibited higher IgY concentrations, leukocyte counts, heterophil levels, and monocyte counts compared to their IPB-D1 progenitors. However, ND antibody titers in IPB-D2 chickens were lower than the protective threshold. Significant positive correlations were observed between leukocytes and lymphocytes, and negative correlations were identified between heterophils and lymphocytes.

**Conclusion:**

IPB-D2 chickens demonstrated a well-balanced immune system with enhanced non-specific (leukocyte and heterophil) and specific (IgY) immune responses. These traits highlight their potential as a disease-resistant poultry line. Future research should focus on molecular selection through transcriptomic analysis to identify candidate genes associated with disease resistance traits, enabling further genetic improvements.

## INTRODUCTION

Local chickens in Indonesia are different from genetically engineered commercial breeds, such as those of Cobb, Lohmann, Ross, and Hubbard [1]. Local chickens typically serve dual purposes as producers of meat and eggs and are mostly raised in rural communities for supplementary enterprises that produce meat and eggs as a source of family nutrition and income. In 2019, the population of local chickens was 301,761,386, which contributed 8.33% to the national meat production [2]. Notably, local chickens have several limitations, such as low productivity [3, 4] and vulnerability to infections, such as Newcastle disease (ND) [5, 6]. These limitations arise from the practice of raising local chickens in open spaces with limited resources.

Recently, local breeders have established breeding and selection programs for local chickens to meet the increasing demand for local chicken products. The IPB-D1 chicken, a newly developed breed of local Indonesian chicken, is a crossbreed of a male F1 hybrid of Pelung and Sentul chickens and a female F1 hybrid of Kampung and Cobb broiler chicken parent stocks [7]. The IPB-D2 chicken is a potential candidate line derived from the IPB-D1 breed. The selection of IPB-D2 chickens was based on multiple immunocompetence characteristics, including immunoglobulin Y (IgY) concentration and ND antibody titers. Indonesia is at risk of ND virus infection, that highly contagious disease that threatens poultry health and causes significant economic losses for farmers. ND can result in mortality rates ranging from 90% to 100% [5]. The primary objective of introducing IPB-D2 chickens was to allow small farmers to raise local chickens with improved disease resistance traits, thereby decreasing disease incidence and mortality [8].

According to our review of the literature, no studies on immunological parameters have been reported. However, studies have investigated specific facets of immunity in IPB-D2 chickens, including the quantification of CD4 and CD8 cells [9], and studies on immunogenetics in IPB-D2 chickens have also been conducted [10–12]. These knowledge gaps have impeded our capacity to define disease resistance characteristics in IPB-D2 chickens and enhance strategies for breeding and management.

This study aims to bridge this knowledge gap by systematically evaluating the disease resistance traits of IPB-D2 chickens. By assessing key immunological parameters and exploring their interrelationships, this research seeks to establish a robust foundation for the development of disease-resistant poultry lines. Ultimately, these efforts aim to support the genetic diversification of local chicken breeds and enhance the sustainability of the poultry industry in Indonesia.

## MATERIALS AND METHODS

### Ethical approval

The study was conducted in compliance with the approval of the Animal Ethics Commission of IPB University (approval number: 224-2021 IPB).

### Study period and location

The study was conducted in November 2021. IPB-D2 chickens were maintained in the field laboratory of the Faculty of Animal Science, IPB University. Leukocyte profiling and differentiation analyses were conducted at the Physiology Laboratory of the School of Veterinary Medicine and Biomedical Sciences, IPB University. IgY measurements and ND antibody titer analyses were performed at the Immunology Laboratory of the School of Veterinary Medicine and Biomedical Sciences, IPB University.

### Experimental birds

One hundred of one-day-old IPB-D2 chickens (40 males and 60 females) were raised in an intensive care system. The chicken population was developed and raised by the Faculty of Animal Science, IPB University. The chickens were housed in enclosures equipped with feeders, drinking water, and egg-laying facilities. Chickens were fed twice daily. Chickens aged up to 4 weeks were provided 100% commercial feed. Chickens aged 5–12 weeks of age received a 70:30 ratio of commercial feed to rice bran. Chickens aged 13–21 weeks were fed a 60:40 mixture of commercial feed and bran. Water was provided *ad libitum*. In 3-day-old IPB-D2 chickens, live ND vaccine (Medivac ND Hitchner B1, Bandung, Indonesia) was administered through eye drops. At 3 weeks of age, the IPB-D2 chickens received another live ND vaccine (Medivac ND La Sota, Bandung, Indonesia) through eye drops. At 12 weeks of ages, a live ND vaccine (Medivac La Sota, Bandung, Indonesia) was administered to IPB-D2 chickens by mixing it with their drinking water.

### Sampling

Blood samples (3 mL) from 100 chickens were collected from 21-week-old IPB-D2 chickens. Subsequently, 1.5 mL of blood was kept in an ethylenediaminetetraacetic acid (EDTA) tube (OneMed, Indonesia) for leukocytes profile and differentiation analysis. The remaining 1.5 mL of blood was stored in a 2 mL tube, for separation of blood serum for analysis of IgY concentration and ND antibody titer.

### Measurement of IgY concentration

The IgY concentration was quantified using an indirect enzyme-linked immunosorbent assay method adapted from Kohl and Ascoli [13], with modifications to the volume of wash and blocking buffers, concentration of the coated antibody, type of stop solution, and wavelength of the microplate reader. After sterilization with ultraviolet light, the microplates were coated with immunoglobulin G (IgG) goat anti-IgY (SAB3700195, Sigma-Aldrich) as a capture antibody at a concentration of 2.5 μg/mL. Antibodies were diluted with buffered bicarbonate (0.005 M carbonate bicarbonate) at pH 9.6 and microplates were incubated overnight at 4°C. Microplates were cleaned 3 times using phosphate-buffered saline and tween-20 (PBST-20, pH 7.4). Subsequently, the microplates were subjected to blocking with 2% bovine serum albumin (BSA), amounting to 100 μL for each well) and then incubated at 37°C for 1 h. The microplate was then cleaned 3 times with 0.05% PBST. Samples of serum were added up to 100 μL (diluted at a ratio 1:100) and incubated at 37°C for 1 h.

After incubating the samples and rinsing with PBST 0.05% 3 times, 100 μL secondary antibody IgG rabbit anti-IgY (A9046, Sigma-Aldrich) was added to each well. Microplates were incubated at 37°C for 1 h and rinsed thrice with 0.05% PBST. In the final reaction, 100 μL tetramethylbenzidine substrate (T0440, Sigma-Aldrich) was added in the dark. The reaction stopped after H_2_SO_4_ was added 10–15 min after the substrate was added. The introduction of H_2_SO_4_ resulted in a yellow change in color, indicating the cessation of the reaction. Optical density was measured at 650 nm using a microplate reader (Bio-Rad).

### ND antibody titers

The hemagglutination inhibition (HI) test was used to evaluate the ND antibody titer, as described by the OIE [14]. Before the HI test, a (hemagglutination) test was administered. HA titer denotes the maximum dilution at which red blood cells exhibited agglutination. Titration was performed at maximum dilution, which resulted in the absence of streaming in the HA solution. This is comparable to the case of a single HA Unit (HAU). A volume of 25 μL PBS was added to each well of a V-bottomed plastic microtiter plate, after which 25 μL serum was added to the first well of the plate. Serial dilutions of serum were made by decreasing the volume to 25 μL and performing twofold dilutions across the plate. Subsequently, 25 μL 4HAU ND antigen was added to each well, and the plate was incubated at room temperature (25°C) for a minimum of 30 min. After incubation, 25 μL of 1% chicken red blood cell solution was added to each well. After gentle agitation, red blood cells were allowed to sediment for approximately 40 min at 25°C. The HI titer was the most diluted serum sample that fully inhibited 4HAU of the antigen. Agglutination was assessed by plate titration. The accuracy of the data was assessed by juxtaposing it with a negative control serum sample, which should exhibit a titer of 22, and a positive control serum sample, which should exhibit a titer within one dilution of the established titer.

### Leukocyte profile and differentiation analysis

Analysis of leukocyte profiles and differentiation was performed using the Giemsa method. A total of 20 µL of EDTA blood was diluted in 380 mL Turk’s solution: 1% gentian violet solution in 1% water, 1 mL glacial acetic acid, and 100 mL distilled water. Subsequently, they were mixed using hand motions executed in a figure-eight pattern. The liquid that was shaken inadequately was discarded.

Subsequently, blood samples were placed in a Neubauer hemocytometer (Assistant, Germany) and allowed to rest until the liquid settled. Leukocyte counts were determined using a biological microscope (Olympus CX33, Japan) at a magnification of 1000×.

Leukocyte differentiation (lymphocytes, heterophils, monocytes, and basophils) was assessed by preparing smear slides. After applying methyl alcohol to the slide for 5 min, the slide was air-dried. After submerging in Giemsa dye for 30 min, the sample was rinsed under running tap water to remove excess dye. The smeared slides were observed under a biological microscope (Olympus CX33, Japan) at 1000× magnification. Numbers of heterophils, monocytes, lymphocytes, and basophils were counted for a total of 100 leukocytes.

### Statistical analysis

Pearson’s correlation test was conducted to evaluate relationships between immunological parameters and disease resistance traits. Data were analyzed using RStudio software (version 4.2.2, https://cran.r-project.org/bin/windows/base/), with significance thresholds set at p < 0.05. Before analysis, data were assessed for normality and linearity to ensure compliance with the assumptions of Pearson’s correlation test. Missing or incomplete data were excluded from the analysis.

## RESULTS

### Disease resistance parameters

Table 1 [7, 15–17] presents the results of disease resistance parameters in IPB-D2 chickens compared with those of other chickens, such as IPB-D1 and Kampung chickens, and commercial laying hens. IPB-D2 chickens had a higher concentration of IgY and heterophil monocytes than parent IPB-D1 chickens.

**Table 1:** Disease resistance parameters in the IPB-D2 chickens.

Parameters	IPB-D2 chicken	IPB-D1 chicken^a^	Kampung chicken^c^	Commercial laying hen^c^	Normal range^d^
IgY concentration (mg/mL)	12.60 ± 1.96	8–11^a^			
ND antibody titer (log_2_ HI unit)	1.55 ± 1.4	> 3^a^			
Leukocyte (10^3^×mm-^3^)	15.29 ± 4.28	11.68 ± 0.88^b^	22.24 ± 6.11^c^	27.34 ± 4.88	20.00–30.00
Lymphocyte (%)	50.96 ± 11.91	65.2 ± 18.20^b^	50.93 ± 12.84	21.06 ± 9.02	55.00–60.00
Heterophil (%)	42.76 ± 12.23	30.71 ± 18.13^b^	40.02 ± 10.66	71.55 ± 10.37	25.00–30.00
Monocyte (%)	4.85 ± 0.95	4.00 ± 4.48	5.91 ± 3.42	6.17 ± 2.50	10.00
Eosinophil (%)	1.61 ± 0.9	1–2	3.14 ± 0.41	1.22 ± 0.16	3.00–8.00
Basophil (%)	Not found	Not found	Not found	Not found	-

a: Al-Habib *et al*.[15] b: Sumantri *et al*.[7] c: Ulupi and Ihwantoro[16] d: Dukes and Swenson [17]. IgY=Immunoglobulin Y

### Correlations of the disease-resistance parameters

**Figure 1:**
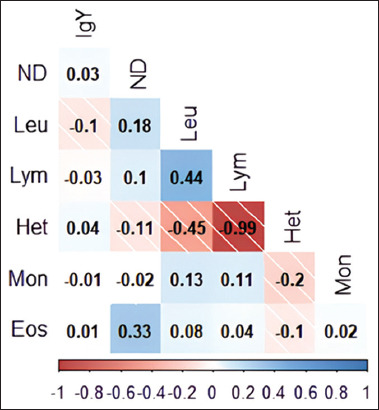
Pearson’s correlation between disease resistance parameters in IPB-D2 chicken. ND=Newcastle-disease antibody titer, IgY=IgY concentration, Leu=Leukocytes, Lym=Lymphocytes, Het=Heterophil, Mon=Monocyte, Eos=Eosinophil.

Pearson’s correlation was used to analyze the relationship between disease resistance parameters in IPB-D2 chickens (Figure 1). In Figure 1, a significant correlation (p < 0.05) is depicted in blue for positive correlations and in red for negative correlations, with values approaching 1 indicating stronger correlations. The most robust positive association was observed between leukocytes and lymphocytes, whereas the most significant negative correlation was observed between heterophils and lymphocytes.

## DISCUSSION

### Disease-resistance parameters

Specific immune systems react with antigens through antigen-antibody interactions. IgY is a primary antibody that is synthesized by chickens in response to infection. This antibody is found in the blood and transmitted to chicks through inheritance. A previous study showed that the serum IgY concentration in chickens ranged between 5 and 15 mg/mL [18]. The mean IgY concentration in IPB-D2 chickens was 12.60 ± 1.96 mg/mL, surpassing that of the progenitors, IPB-D1 chickens, which ranged from 8 to 11 mg/mL [15]. Because IPB-D2 chickens exhibited higher IgY concentrations, they were selected from IPB-D1 chickens based on this criterion.

Antibody titers are crucial for assessing protective immunological responses to ND [19]. Ghaniei and Mohammadzadeh [20] classified an ND antibody titer ≥4 log2 HI units as protective. In the meantime, an ND antibody titer of <3 log2 HI units was classified as non-protective. In IPB-D2 chickens, the ND antibody titer was 1.55 ± 1.40 log2 HI. The ND antibody titer in the IPB-D2 chickens was lower than that in the IPB-D1 chickens. Both were classified as non-protective.

The decrease in ND antibody titer in IPB-D2 chickens could be due to low exposure to ND virus in the environment. Rahman *et al*. [19] suggested that a low ND antibody titer arises from ineffective immunization or maternal antibodies that neutralize the virus. Inadequate ND antibody titers may result from vaccination. Vaccines containing live attenuated viruses are referred to as live vaccinations. Live vaccines elicit mucosal surface-specific immunity. Vaccination elicited an IgA-mediated mucosal response [21].

ND is one of the most prevalent animal diseases worldwide. Hartaputera *et al*. [6] reported that ND is endemic to Penebel, Tabanan, Bali Province, Indonesia. The elevated ND incidence on farms, particularly local poultry farms, is attributed to insufficient disease awareness among farmers. ND is induced by the ND virus a member of the family Paramyxoviridae in the genus *Avulavirus*. ND causes damage to the respiratory, digestive, and neurological systems. In general, ND leads to immunosuppression and mortality in chickens.

Antibodies are one of the indicators of immunocompetence in chickens. Antibody production in chickens involves leukocyte differentiation. Kolesnik *et al*. [22] demonstrated that leukocytes play a role in specific and non-specific immune systems by producing antibodies and phagocytizing. Tigner *et al*. [23] demonstrated that leukocyte differentiation involves granulocytes (heterophils, eosinophils, and basophils) and agranulocytes (lymphocytes and monocytes). IPB-D2 chickens had a higher total leukocyte count than IPB-D1 chickens. Compared with Kampung chickens and commercial laying hens, the leukocyte count of IPB-D2 chickens was lower than that of IPB-D1 chickens and was below the reference range (Table 1) [7, 15–17]. Kampung chickens and commercial laying hens may be infected by certain pathogens. An elevated leukocyte count indicates humoral and cellular immune responses to pathogens [24].

Lymphocytes orchestrate immune responses in higher organisms. Like other vertebrates, chicken lymphocytes are categorized as B- or T-lymphocytes according to their origin and function [25]. The lymphocyte count in IPB-D1 chickens was higher than that in IPB-D2 chickens, the latter of which was equivalent to that of Kampung chickens but higher than that of commercial-laying chickens.

Wang *et al*. [26] demonstrated that heterophils serve as an initial defense against pathogens that can lead to infection or inflammation. IPB-D2 chickens had a total heterophil count comparable with that of the Kampung chickens but surpassed that of the IPB-D1 chickens. Compared with that of commercial laying chickens, the heterophil count of IPB-D2 chickens was reduced. Zmrhal *et al*. [27] reported that an elevated heterophil count in chickens suggests exposure to bacterial or viral infections or physiological stress.

Monocytes constitute the subsequent line of defense that the body employs against infections. Monocytes function as macrophage precursors [28]. Sufiriyanto *et al*. [29] demonstrated that monocytes can differentiate into macrophages on encountering pathogens in the tissues of the body. The monocyte counts of the IPB-D2 chickens exceeded that of the IPB-D1 chickens. Infection with pathogenic agents is likely responsible for elevated monocyte counts in IPB-D2 chickens. This aligns with monocyte functions.

Eosinophils are polymorphonuclear granulocytes in the spinal cord, which eventually enter the bloodstream. The typical lifespan of eosinophils is 3–5 d. Hidayat *et al*. [30] demonstrated that eosinophils have two primary functions: to attack and eradicate pathogenic microorganisms and to produce enzymes that mitigate inflammation. The eosinophil count of the IPB-D2 chickens was comparable to that of the IPB-D2 chickens and commercial laying hens but lower than that of Kampung chickens.

White blood cells participate in allergic responses. Basophils are white blood cells containing histamine, bradykinin, serotonin, and lysosomal enzymes that initiate inflammatory responses. Dukes and Swenson [17] reported that the proportion of basophils in the blood is <1%. In this study, the calculation findings indicated that basophils were absent in the blood of IPB-D2, IPB-D1, and Kampung chickens and commercial laying hens. This result does not indicate an absence of basophils in IPB-D2 chickens. The scarcity of basophils, comprising 0.07% of the bloodstream, is noteworthy [31].

### Correlations of the disease-resistance parameters

The most robust positive relationships were identified between leukocytes and lymphocytes and between eosinophils and ND antibody titers, with correlation coefficients of 0.44 and 0.33, respectively. Ikhwan *et al*. [32] classified correlation values of 0.2–0.4 as weak and correlation values of 0.4–0.6 as moderate. This study identified the largest negative correlations between heterophils and leukocytes, with a correlation value of −0.45, and between heterophils and lymphocytes, with a correlation value of −0.99. Lymphocytes and heterophils are the two most abundant types of leukocytes and have different immunological functions. Through antibody production, lymphocytes play a crucial role in cell-mediated adaptive immunity (T cells) and humoral adaptive immunity (B cells). Heterophils are phagocytic cells of the innate immune system that serve as primary defenses against bacterial infections. According to our literature review, this is the first study to report the relationship between disease resistance characteristics in Indonesian local chickens, particularly IPB-D2 chickens.

The IPB-D2 chicken line can potentially develop into a disease-resistant chicken lineage. IPB-D2 propagation can be achieved by mating IPB-D2 chickens with elevated IgY concentrations, suggesting that the progeny will exhibit enhanced disease resistance.

## CONCLUSION

The IPB-D2 chicken line exhibits significant advancements in immune system characteristics compared to its IPB-D1 progenitors, including higher IgY concentrations and improved leukocyte differentiation. These enhancements reflect a well-regulated balance between specific and nonspecific immune responses, suggesting that IPB-D2 chickens are better equipped to handle disease challenges. However, the observed lower ND antibody titers highlight a need for optimized vaccination protocols to strengthen protective immunity. The findings of this study underline the potential of IPB-D2 chickens as a sustainable, disease-resistant poultry line suitable for local farming systems in Indonesia.

Future research should focus on exploring the molecular mechanisms underlying these immune traits, particularly through transcriptomic and genomic analyses, to further refine breeding strategies. These efforts will contribute to the genetic diversification and sustainability of the poultry industry while addressing the growing demand for resilient and productive chicken breeds.

## AUTHORS’ CONTRIBUTIONS

DL: Conducted the study, interpreted and analyzed the data, and drafted and revised the manuscript. SM: Developed the study concept, conducted the investigation, and supervised the study. NU: Conducted the validation and oversight of the study and reviewed the manuscript thoroughly. AG: Validated and supervised the study and revised the manuscript. IK: Conducted the data validation and reviewed and revised the manuscript. AA: Conducted thorough validation, review, and manuscript editing. CS: Developed the study framework, confirmed its validity, explored research aspects, and supervised the study. All authors have read and approved the final version of the manuscript.
